# The up-regulation of Myb may help mediate EGCG inhibition effect on mouse lung adenocarcinoma

**DOI:** 10.1186/s40246-016-0072-4

**Published:** 2016-07-25

**Authors:** Hong Zhou, Joseph Manthey, Ekaterina Lioutikova, William Yang, Kenji Yoshigoe, Mary Qu Yang, Hong Wang

**Affiliations:** 1 Department of Mathematical Science, School of Health and Natural Sciences, University of Saint Joseph, 1678 Asylum Avenue, West Hartford, CT 06117 USA; 2 Joint Bioinformatics Ph.D. Program, University of Arkansas at Little Rock and University of Arkansas for Medical Sciences, 2801 S. University Avenue, Little Rock, AR 72204 USA; 3 Susan L. Cullman Laboratory for Cancer Research, Department of Chemical Biology and Centre for Cancer Prevention Research, Ernest Mario School of Pharmacy, Rutgers, The State University of New Jersey, 164 Frelinghuysen Road, Piscataway, NJ 08854 USA

## Abstract

**Background:**

Green tea polyphenol epigallocatechin-3-gallate (EGCG) has been demonstrated to inhibit cancer in experimental studies through its antioxidant activity and modulations on cellular functions by binding specific proteins. By means of computational analysis and functional genomic approaches, we previously identified a set of protein coding genes and microRNAs whose expressions were significantly modulated in response to the EGCG treatment in tobacco carcinogen-induced lung adenocarcinoma in A/J mice. However, to what degree these genes are involved in the cancer inhibition of EGCG remains unclear.

**Results:**

In this study, we further employed statistical methods and literature research to analyze these data in combination with The Cancer Genome Atlas (TCGA) lung adenocarcinoma datasets for additional data mining. Under the assumption that, if a gene mediates EGCG’s cancer inhibition, its expression level change caused by EGCG should be opposite to what occurred in the carcinogenesis, we identified Myb and Peg3 as the primary putative genes involved in the cancer inhibitory activity. Further analysis suggested that the regulation of Myb could be mediated through an EGCG-upregulated microRNA, miR-449c-5p.

**Conclusions:**

Although the actions of EGCG involve multiple targets/pathways, further analysis by mining the existing genomic datasets revealed that the upregulations of Myb and Peg3 are likely the key anti-cancer events of EGCG in vivo.

**Electronic supplementary material:**

The online version of this article (doi:10.1186/s40246-016-0072-4) contains supplementary material, which is available to authorized users.

## Background

Green tea polyphenol epigallocatechin-3-gallate (EGCG) has been shown to have preventive effect for several diseases including cancer [1–4]. A substantial number of studies have been conducted to uncover the cancer preventive mechanisms of EGCG at the cellular and molecular levels including experimental studies using animal models. The results from these studies support that the treatment with EGCG or EGCG-rich tea extract leads to a wide range of responses and that the cancer prevention activities are most likely to be mediated through multiple mechanisms resulted from direct scavenging of stress molecules such as reactive oxygen species (ROS) and/or the physical interactions of EGCG with specific proteins to modulate gene expression and cellular signaling. However, most experimental evidence supporting anti-cancer mechanisms of EGCG are obtained from in vitro studies. Whether or not these mechanisms play significant roles in the cancer prevention/inhibition in vivo remains to be determined.

The tobacco carcinogen-induced lung carcinogenesis in A/J mice is a well-characterized animal model. It has been shown using this animal model that tumor multiplicity and size are effectively inhibited when mice are fed an experimental diet containing EGCG [2, 5]. Using this animal model, our recent study has demonstrated that cellular changes in lung cancer cells treated with EGCG are associated with alterations in both messenger RNA (mRNA) and microRNA (miRNA) expressions [6]. Computational analysis on microarray data revealed that the EGCG-induced expression changes of some genes also involved miRNA-mediated gene regulation [6].

It is well-recognized that EGCG treatment can cause a wide range of responses at both the cellular and molecular levels [2]. Using “EGCG” as the keyword to search through NIH’s TOXNET databases, specifically the Comparative Toxicogenomics Database (CTD) which provides scientific data describing relationships between chemicals, genes, and human diseases, we found that EGCG treatment or co-treatment with other agents can impact the expression of about 2000 genes in both humans and mice. Our recent study also showed that, in the carcinogen-induced lung tumor in A/J mice, EGCG treatment induces the expression level change of 367 genes (at least onefold change) [6]. Since the large group of genes up- or down-regulated by EGCG includes genes which are not related to lung carcinogenesis [7, 8], identifying the relevant genes would advance our understanding of the cancer inhibition mechanism of EGCG.

In this study, we employed statistical approaches and literature research and combined the data from The Cancer Genome Atlas (TCGA) [9] to further determine the candidate genes, including miRNA, which participate in the EGCG inhibition mechanism. We focused on exploring the lung cancer-related genes and identifying the ones that could potentially play predominant roles in mediating the cancer inhibition by EGCG in the carcinogen-induced A/J mouse lung cancer model.

## Methods

Microarray data of mRNA expression and miRNA expression were obtained from two sources. The first was from our previous study that compared the mRNA and miRNA expression profiles in A/J mice bearing 4-(methylnitrosamino)-1-(3-pyridyl)-1-butanone (NNK)-induced lung tumor fed on the diet containing 0.4 % EGCG or the control AIN93M diet [6]. In this dataset, mRNA expression profiles include 3 microarray results of lung tumors collected from 3 control mice and 3 EGCG-treated mice, and the miRNA expression profiles include microarray results of lung tumors collected from 8 control mice and 8 EGCG-treated mice. The second source was the gene expression profile batch 37 of lung adenocarcinoma obtained from TCGA data matrix [9]. The filter settings used to obtain this dataset include Data Level = 3, Availability = Available, Center/Platform = All, Access Tier = Public, and Tumor/Normal = Tumor-matched.

When performing statistical analysis on the microarray datasets, we made the following assumptions.

1.  There are random variations in gene expression among samples which are independent of the treatments. These random variations in gene expression represent the normal variations of individuals in a group of experimental animals.
2.  When treated with EGCG, the expression changes of responsive genes are expected to be statistically significant and consistent among all treated samples compared to the control.

To analyze the mRNA expression profiles, within the 3 controls and the 3 EGCG-treated samples, we first removed those genes whose expression in the 3 EGCG treatments were not consistently up or down compared to the average of the 3 controls. This means that we only selected those genes whose expressions were consistently up-regulated or down-regulated in all the 3 EGCG-treated samples compared to the average of the 3 controls. Then, we computed the difference between the averaged expression levels of the 3 controls and the 3 treatments for each gene. The standard deviation was calculated for the difference distribution, and z-scores were obtained for each gene. Only those genes whose z-scores were ≥3.00 or ≤−3.00 were considered genes that were impacted significantly by EGCG treatment.

To analyze the miRNA profiles, with the 8 controls and 8 EGCG treatments, the difference between averaged control and treatment was obtained for each miRNA. The standard deviation was calculated for the differences, and z-scores were obtained for each miRNA. We first selected those miRNAs whose z-scores were ≥2.00 or ≤−2.00. Then, among those selected miRNAs, we conducted an independent sample *t* test between the 8 controls and the 8 EGCG samples. Only those miRNAs with *p* values of the *t* test less than 0.05 were considered as miRNAs that were responsive to EGCG treatment.

From TCGA database, we obtained microarray gene expression data for 21 human lung adenocarcinoma samples (batch 37) publically released by Michael Topal and Katherine Hoadley from the University of North Carolina at Chapel Hill on February 10, 2009 [9]. These 21 datasets represent the normalized gene expression differences between lung adenocarcinoma and a normal cell line. Although there are another 11 sets of such data in batch 52, some gene expressions are null in batch 52, and batch 52 was not used in this study. After the standard deviation was calculated based on the average of the 21 datasets, z-scores were computed for each gene. Only those genes whose expression levels are all up or all down in the 21 sets and whose z-scores were ≥3.00 or ≤−3.00 were selected.

Gene pathway analyses were conducted using iPathwayGuide (https://apps.advaitabio.com/ipg/home). The only adjustable setting of iPathwayGuide is its DE threshold. The settings used were as follows: Fold Change (log) = 1.0 and Adjusted *p* value = 0.01. Gene interaction analysis was conducted using the PCViz web tool http://www.pathwaycommons.org.

## Results

In the comparison of the microarray results of mRNA expression profiles of the 3 controls and 3 EGCG samples, mRNAs whose expression levels are not consistently up or down between the controls and EGCG-treated samples were removed. We selected only these consistent data records because they tend to be more reliable. Fourteen thousand seven hundred sixty-five records were selected. Z-scores were computed for every mRNA (gene). Only those genes whose z-scores were ≥3.00 or ≤3.00 were considered genes that are impacted significantly by EGCG treatment, i.e., the expression changes of these genes between the control and the sample are likely a result of the EGCG treatment. There were 225 unique genes selected (see Additional file 1). The large number of genes impacted by EGCG treatment is expected as EGCG treatment induces a wide range of biological responses [1–3].

In the A/J mouse lung carcinogenesis model, the tumor multiplicity and size are effectively inhibited when mice are fed a diet containing EGCG [2, 5]. Apoptosis was induced and pro-proliferation signalings were reduced in tumors after a long-term treatment with EGCG [10, 11]. The gene expression profiles showed that cell cycle regulation and inflammation were the most impacted pathways by a long-term EGCG treatment (~20 weeks) [12]. Since a long-term EGCG treatment may cause multiple waves of responses which can disguise the targeted genes or pathways of EGCG, we preferred a short-term treatment experiment for elucidating the earlier responses, as described in our recent study in which EGCG was applied for only 1 week [6]. Although it is somewhat surprising that none of the 17 signature genes identified by Lu et al. [12] were found among the 225 genes, such a discrepancy indicates that the short-term treatment causes a wider range of early responses while the result induced by the long-term treatment represents the overall consequence post the early response. Thus, the result of short treatment offers better chance to identify the mechanism.

By analyzing the 225 genes using iPathwayGuide, the only pathway approaching statistical significance (*p* = 0.08) is the Kyoto Encyclopedia of Genes and Genomes (KEGG) chemokine pathway [13]. This pathway is associated with multiple cellular functions such as cellular growth and differentiation, cell survival, migration, apoptosis, chemotaxis, and ROS production [14–26]. Indeed, this is consistent with what was reported previously, either with a long-term or short-term EGCG treatment [6, 10–12]. This result supports that the anti-cancer mechanism of EGCG is mediated through the regulation of cellular growth, apoptosis, differentiation, and migration.

By applying the same statistical selection process on the 21 lung adenocarcinoma datasets collected from the TCGA database, 478 genes were selected from the TCGA LUAD datasets since they displayed significantly up-regulated or down-regulated expression levels in the lung adenocarcinoma tissues. Comparing with the 225 genes selected from EGCG treatment, 16 genes were found to be in both groups. We postulated that if EGCG is truly inhibiting the lung carcinogenesis, then it should reverse the expression levels of some genes that were over-expressed or under-expressed in lung cancer tissues. Thus, we were searching for those genes whose expression levels were reversed in the EGCG-treated samples when compared with the TCGA LUAD datasets. In the overlapped genes between the 478 genes selected from the TCGA LUAD datasets and the 225 genes selected from EGCG-treated group, only Myb, Peg3, and Myl4 display a reverse expression pattern (Table 1). While Myl4 was not found to be related to carcinogenesis based on the existing literature, both Myb and Peg3 were reported to be involved in carcinogenesis [27–30]. Although Myb has been considered as an oncogene [31, 32], its down-regulation in human lung cancer suggests that it may have dual functions. Nevertheless, this comparison suggested that EGCG’s inhibition on mouse lung carcinogenesis reversed the carcinogenesis-associated changes, and Myb and Peg3 are the putative genes that mediate such inhibition effect.

**Table 1:** Genes whose expression levels were significantly altered and were found in both the EGCG experiment and the TCGA dataset

Genes	From normal to LUAD	From control to EGCG treatment
Cox7a1	Up	Up
Myh11	Up	Up
Fmo3	Up	Up
Myb	Down	Up
Cxcl13	Up	Up
Scgb3a1	Up	Up
F13a1	Up	Up
Peg3	Down	Up
Acta2	Up	Up
Fabp4	Up	Up
Clic6	Up	Up
Myl4	Down	Up
S100a9	Up	Up
Col8a2	Up	Up
Bank1	Up	Up
Mmp9	Up	Up

Using the functional annotation tool of David Bioinformatics Resources 6.7 (http://david.abcc.ncifcrf.gov/) to analyze these overlapped genes also identified F13a1, Fmo3, Mmp9, and Myh11 as related to cancer. Among these genes, Mmp9 is specifically related to lung cancer [33–36]. However, these genes including Mmp9 are less likely to be related to the inhibition function of EGCG since they do not exhibit a reversed expression pattern.

**Fig. 1:**
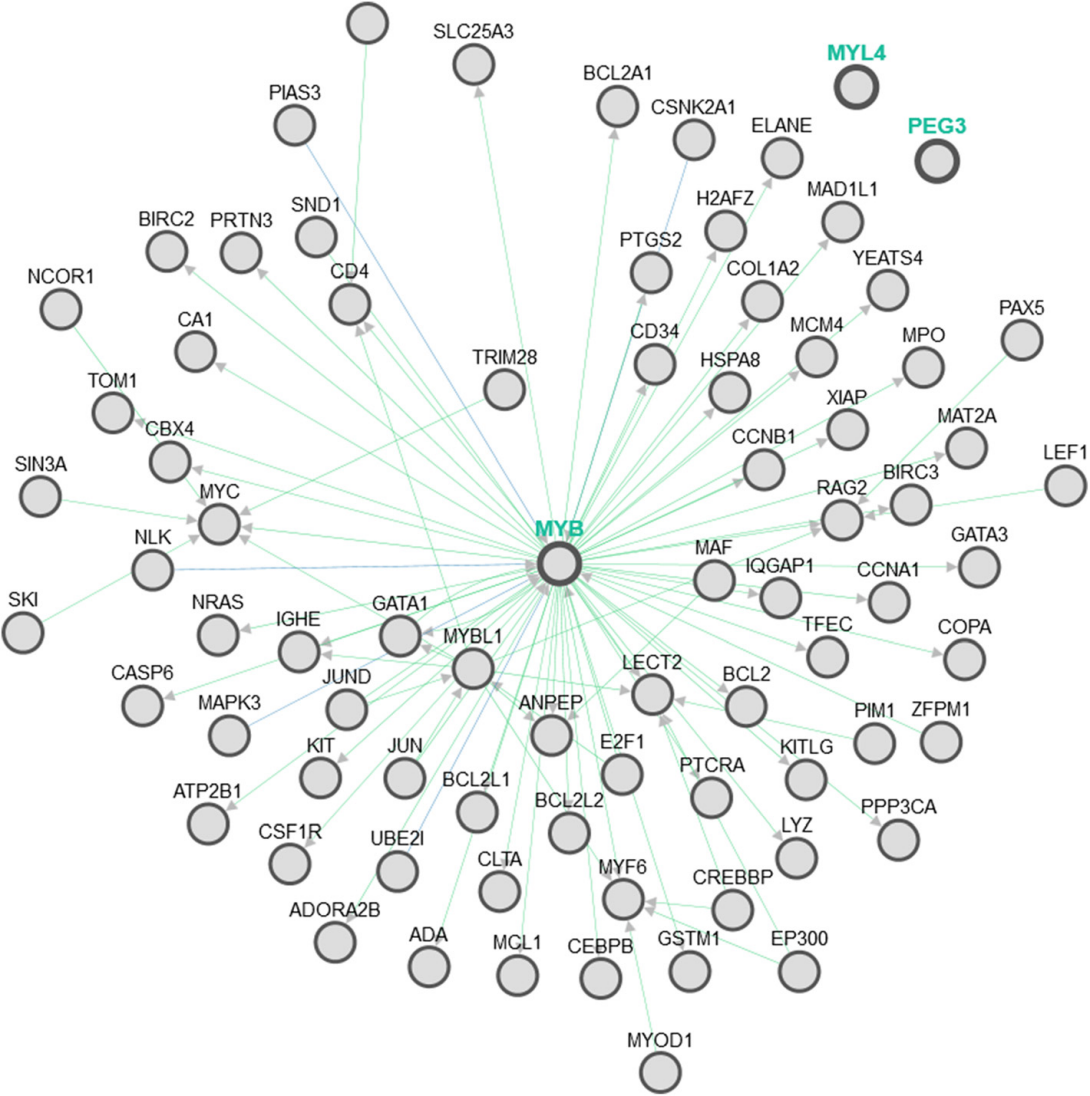
The gene interaction network of Myb, Peg3, and Myl4. There are 76 genes and 88 interactions for Myb, while none for Peg3 or Myl4

In order to assess the possible roles of Myb, Peg3, and Myl4, we employed the PCViz network visualization tool (www.pathwaycommons.org), a web tool to construct gene interaction network based on information mined from published literature and to analyze the possible gene interactions for Myb, Peg3, and Myl4. We found that there are no identified gene interactions for both Peg3 and Myl4, while Myb has the potential to control the expression of a large number of genes including Myc, Nras, and Bcl2 that are well-characterized for their role in tumorigenesis (Fig. 1). This suggests that Myb might play a much more critical role than Peg3 in mediating the anti-cancer activity of EGCG.

A further analysis on those genes that were identified by the PCViz network to be affected by Myb shows that there are 29 mRNA genes whose expressions were affected by EGCG treatment. However, there are only 3 genes, Col1a2, Ccnb1, and Copa, whose expression levels could be considered to be significantly impacted by EGCG treatment. Col1a2 was up-regulated by 1.8-fold, while Ccnb1 and Copa were down-regulated by 2.4- and 3.4-fold, respectively. Clearly, much more studies are required before any conclusion can be drawn on how Myb mediates EGCG’s anti-cancer activities.

In our previous study, we demonstrated that miRNAs can mediate the gene regulation by EGCG [6]. Here, we used statistical methods to further investigate the possible roles of miRNA in EGCG treatments, especially if miRNA can possibly mediate the regulation of Myb and/or Peg3 by EGCG. We first used statistical methods to identify those miRNAs whose expression levels were significantly modified by EGCG treatment. The selection of such miRNAs is a two-step process. In step 1, the averaged miRNA expression levels were computed for both the 8 controls and 8 samples. The averaged difference was then calculated for each miRNA. By treating the pool of the averaged differences as normally distributed, z-scores were computed for each miRNA. Only those miRNAs for which |*z*| ≥ 2.00 were selected. In step 2, an independent sample *t* test (two tails) was conducted between the 8 controls and 8 EGCG-treated samples for each selected miRNA. Those miRNAs that did not show a statistically significant difference (*p* ≤ 0.05) between the controls and samples were removed. Using this selection process, 27 miRNAs were selected as shown in Table 2.

**Table 2:** miRNAs that display a statistically significant difference between the controls and EGCG treatments

Up-regulated miRNAs	Down-regulated miRNAs
mmu-miR-2137	mmu-miR-1199-3p
mmu-miR-5131	mmu-miR-218-5p
mmu-miR-680	mmu-miR-7a-5p
mmu-miR-5130	mmu-miR-374c-5p
mmu-miR-144	mmu-miR-449c-5p
mmu-miR-486	mmu-miR-450a-2-3p
mmu-miR-711	mmu-miR-696
mmu-miR-449a	
mmu-miR-193	
mmu-miR-34c-5p	
mmu-miR-2861	
mmu-miR-211-3p	
mmu-miR-763	
mmu-miR-128-2-5p	
mmu-miR-542-5p	
mmu-miR-3473	
mmu-miR-511-3p	
mmu-miR-34c-5p	
mmu-miR-143-5p	
mmu-miR-5115	

Among the 27 miRNAs, 20 were up-regulated. Since the function of miRNA is to suppress the maturation of its targeting mRNAs, up-regulated expression of miRNA usually result in down-regulated mRNA expression and vice versa. Using both Diana microT-CDS v3.0 and miRDB microRNA target prediction tools, we collected a number of potential microRNA target genes for the 27 miRNAs in Table 2. Based on the up/down-regulated expression levels of these 27 miRNAs, we only selected a protein coding gene as a target of a microRNA if (1) its expression change is opposite to the change of the miRNA expression and (2) it exists in the 225 gene groups. The results are summarized in Table 3.

**Table 3:** Genes regulated by EGCG potentially mediated through miRNA

Targeted gene	Gene expression change	miRNA(s)	miRNA expression change
Rsph4a	Up	mmu-miR-374c-5p	Down
Rbm24	Up	mmu-miR-374c-5p	Down
DCN	Up	mmu-miR-374c-5p	Down
Acta2	Up	mmu-miR-374c-5p	Down
Zfp182	Up	mmu-miR-374c-5p	Down
KLHL4	Up	mmu-miR-374c-5p	Down
ART3	Up	mmu-miR-374c-5p	Down
GAS1	Up	mmu-miR-374c-5p,mmu-miR-449c-5p	Down
Myb	Up	mmu-miR-449c-5p	Down
Elmod1	Up	mmu-miR-449c-5p	Down
Ldb3	Up	mmu-miR-449c-5p	Down
Calcb	Up	mmu-miR-449c-5p	Down
ZFHX4	Up	mmu-miR-449c-5p,mmu-miR-450a-2-3p	Down
IGFBP5	Up	mmu-miR-450a-2-3p	Down
Bnc1	Up	mmu-miR-450a-2-3p	Down
GABRP	Up	mmu-miR-450a-2-3p	Down
Cd19	Up	mmu-miR-450a-2-3p	Down
AQP4	Up	mmu-miR-450a-2-3p	Down
VCAN	Up	mmu-miR-450a-2-3p	Down
Cnr1	Up	mmu-miR-218-5p,mmu-miR-374c-5p	Down
EBF1	Up	mmu-miR-218-5p, mmu-miR-374c-5p	Down
LASP1	Up	mmu-miR-218-5p	Down
Gm3008	Up	mmu-miR-218-5p	Down
Gstm6	Up	mmu-miR-218-5p	Down
BICD1	Up	mmu-miR-218-5p,mmu-miR-374c-5p	Down
ACSL1	Up	mmu-miR-218-5p,mmu-miR-449c-5p	Down
Six4	Up	mmu-miR-218-5p,mmu-miR-7a-5p	Down
AACS	Up	mmu-miR-218-5p,mmu-miR-7a-5p	Down
Pfn2	Up	mmu-miR-7a-5p	Down
Fbp1	Down	mmu-miR-34c-5p	Up

Thirty genes were found to be candidates targeted by the 27 miRNAs. It is interesting to notice that of the 30 genes, only Fbp1 has its expression level up-regulated; and of the 27 miRNAs, only 6 were found to regulate gene expressions significantly. In this candidate gene list, Myb gene is shown as a target of mmu-miR-449c-5p, indicating that the miRNA such as miR-449c-5p may play an important role in the inhibitory activity of EGCG.

## Discussion

Since EGCG treatment causes a wide range of responses at both cellular and molecular levels, it is difficult to elucidate how EGCG unleashes its anti-cancer effects, especially in an in vivo model like the carcinogen-induced lung adenocarcinoma in A/J mice. One possible approach is to apply the genomic studies with bioinformatics analysis integrated with literature research. We demonstrated that this is a feasible approach in this study.

A critical assumption in this study is that most genes have small variations on their expressions as a random phenomenon, while genes impacted by EGCG treatment would exhibit larger variations, i.e., statistically significant variations. Though the distribution of the mRNA expression differences between the averaged control and EGCG treatment is not a normal distribution, the large size of the sample (14,765 gene records) allows us to make this assumption. We made a similar assumption on the miRNA expression alterations caused by EGCG treatment. However, the sample size of miRNAs is only 656, much smaller than that of mRNAs. To ensure the significance of the miRNAs selected from our statistical approach, we used a two-step process. In the first step, z-score of 2.0 was used as the cutoff. A z-score of 2.0 was used instead of a z-score of 3.0 due to the relatively small sample size of miRNA genes. In addition, the independent sample *t* test used in the second step further ensures the significance of the selected miRNAs. After identifying 225 mRNAs and 27 miRNAs whose altered expression levels are statistically significant, we assumed that some of these selected genes, either mRNA or miRNA, may be related to EGCG’s anti-cancer mechanism.

While the pathway analysis showed that this group of 225 genes is likely to be involved in the chemokine pathway which plays an important role in migration, cell growth and differentiation, apoptosis, and chemotaxis [13], it did not reveal the genes related to anti-cancer activity of EGCG. The TCGA lung adenocarcinoma (LUAD) datasets contain the normalized gene expression microarray data which records the fold changes between lung adenocarcinoma tissues and a normal cell line. These datasets serve as a good supplemental control to our experiment. We expect that if a gene mediates the anti-cancer activity of EGCG, then its expression level change in our EGCG treatment experiment should be opposite to its expression level change in the TCGA LUAD datasets. Three genes, Myb, Peg3, and Myl4, were found to match this criterion, and their expression level changes are statistically significant. While our literature research has not found any role of Myl4 in carcinogenesis, Peg3 has been reported to act like a tumor suppressor gene [29, 30].

As a transcription factor, Myb gene has been reported to be involved in the progression of different types of cancer including lung cancer and has recently been considered as an oncogene because of its critical role in cell proliferation and differentiation [27, 28, 31, 32]. However, its expression which is reduced in TCGA LUAD datasets suggests that Myb expression decrement is necessary for human lung carcinogenesis. Although we do not understand the reason, one possibility is that Myb may have dual functions or tissue specific function. If so, our result here supports that Myb is necessary for EGCG to exert its anti-cancer effect. Interestingly, our result further showed that Myb could be regulated by EGCG down-regulated miRNA, miR-449c-5p. Again, if Myb does not act as oncogene but mediates the cancer inhibitory activity of EGCG, this result suggests that miRNA-mediated gene regulation is an important action of EGCG although these miRNAs may not be directly regulated by EGCG as we discussed previously [6].

## Conclusions

EGCG treatment leads to the expression level changes of a large number of genes, causing a wide range of biological responses. However, it is unlikely that majority of these regulated genes mediate the anti-cancer activity of EGCG. Our analysis on the whole transcriptome integrated with TCGA datasets on human lung cancer revealed that EGCG reverses the lung carcinogenesis-associated changes of Myb and Peg3, suggesting that Myb and Peg3 play key roles in the anti-cancer activity of EGCG. In addition, our analysis of the miRNA profiles suggests that the up-regulation of Myb by EGCG could be achieved by EGCG-induced down-regulation of miRNA mmu-miR-449c-5p, supporting a role of miRNA in the anti-cancer activity of EGCG.

## Additional file

Additional file 1: The mRNAs significantly regulated by EGCG in the NNK-induced A/J mouse lung tumor. (36kb xlsx)

## References

[1] Khan N, Afaq F, Saleem M, Ahmad N, Mukhtar H (2006). Targeting multiple signaling pathways by green tea polyphenol (-)-epigallocatechin-3-gallate. Cancer Res.

[2] Yang CS, Wang X, Lu G, Picinich SC (2009). Cancer prevention by tea: animal studies, molecular mechanisms and human relevance. Nat Rev Cancer.

[3] Yang CS, Wang H, Li GX, Yang Z, Guan F, Jin H (2011). Cancer prevention by tea: evidence from laboratory studies. Pharmacol Res.

[4] Kitamura M, Nishino T, Obata Y, Furusu A, Hishikawa Y, Koji T, Kohno S (2012). Epigallocatechin gallate suppresses peritoneal fibrosis in mice. Chem Biol Interact.

[5] Ju J, Lu G, Lambert JD, Yang CS (2007). Inhibition of carcinogenesis by tea constituents. Semin Cancer Biol.

[6] Zhou H, Chen J, Yang CS, Yang M, Deng Y, Wang H (2014). Gene regulation mediated by microRNAs in response to green tea polyphenol EGCG in mouse lung cancer. BMC Genomics.

[7] Kim HS, Quon MJ, Kim J (2014). New insights into the mechanisms of polyphenols beyond antioxidant properties; lessons from the green tea polyphenol, epigallocatechin-3-gallate. Redox Biol.

[8] El-Telbany A, Ma PC (2012). Cancer genes in lung cancer: racial disparities: are there any?. Genes Cancer.

[9] https://tcga-data.nci.nih.gov/tcga/dataAccessMatrix.htm?mode=ApplyFilter, accessed on 13 Nov 2014.

[10] Lu G, Liao J, Yang G, Reuhl KR, Hao X, Yang CS (2006). Inhibition of adenoma progression to adenocarcinoma in a 4-(methylnitrosamino)-1-(3-pyridyl)-1-butanone-induced lung tumorigenesis model in A/J mice by tea polyphenols and caffeine. Cancer Res.

[11] Li GX, Chen YK, Hou Z, Xiao H, Jin H, Lu G, Lee MJ, Liu B, Guan F, Yang Z (2010). Pro-oxidative activities and dose-response relationship of (-)-epigallocatechin-3-gallate in the inhibition of lung cancer cell growth: a comparative study in vivo and in vitro. Carcinogenesis.

[12] Lu Y, Yao R, Yan Y, Wang Y, Hara Y, Lubet RA, You M (2006). A gene expression signature that can predict green tea exposure and chemopreventive efficacy of lung cancer in mice. Cancer Res.

[13] http://www.genome.jp/kegg/pathway.html. Accessed on 24 Nov 2014.

[14] Mellado M, Rodriguez-Frade JM, Mañes S, Martinez-A C (2001). Chemokine signaling and functional responses: the role of receptor dimerization and TK papthway activation. Annu Rev Immunol.

[15] Wong MM, Fish EN (2003). Chemokines: attractive mediators of the immune response. Semin Immunol.

[16] Curnock AP, Logan MK, Ward SG (2002). Chemokine signaling: pivoting around multiple phosphoinostitide 3-kinases. Immunology.

[17] Busillo JM, Benovic JL (1768). Regulation of CXCR4 signaling. Biochim Biophys Acta.

[18] Olson TS, Ley K (2002). Chemokines and chemokine receptors in leukocyte trafficking. Am J Physiol Regul Integr Comp Physiol.

[19] Knall C, Young S, Nick JA, Buhl AM, Worthen GS, Johnson GL (1996). Interleukin-8 regulation of the Ras/Raf/mitogen-activated protein kinase pathway in human neutrophils. J Biol Chem.

[20] Hauck CR, Klingbeil CK, Schlaepfer DD (2000). Focal adhesion kinase functions as a receptor-proximal signaling component required for directed cell migration. Immunol Res.

[21] Chandrasekar B, Bysani S, Mummidi S (2004). CXCL16 signals via Gi, phosphatidylinositol 3-kinase, Akt, I kappa B kinase, and nuclear factor-kappa B and induces cell-cell adhesion and aortic smooth muscle cell proliferation. J Biol Chem.

[22] Schwartzberg PL, Finkelstein LD, Readinger JA (2005). TEC-family kinases: regulators of T-helper-cell differentiation. Nat Rev Immunol.

[23] Ticcioni M, Essafi M, Jeandel PY, Davi F, Cassuto JP, Deckert M, Bernard A (2007). Hemeostatic chemokines increase survival of B-chronic lymphocytic leukemia cells through inactivation of transcription factor FOXO3a. Oncegene.

[24] Johnson Z, Power CA, Weiss C, Rintelen F, Ji H, Ruckle T, Camps M, Wells TN, Schwarz MK, Proudfoot AE, Rommel C (2004). Chemokine inhibition—why, when, where, which and how?. Biochem Soc Trans.

[25] Bach TL, Chen QM, Kerr WT, Wang Y, Lian L, Choi JK, Wu D, Kazanietz MG, Koretzky GA, Zigmond S, Abrams CS (2007). Phospholipase cbeta is critical for T cell chemotaxis. J Immunol.

[26] Hornstein I, Alcover A, Katzav S (2004). Vav proteins, masters of the world of cytoskeleton organization. Cell Signal.

[27] George OL, Ness SA (2014). Situational awareness: regulation of the Myb transcription factor in differentiation, the cell cycle and oncogenesis. Cancers.

[28] Stenman G, Andersson MK, Andrén Y (2010). New tricks from an old oncogene gene fusion and copy number alterations of MYB in human cancer. Cell Cycle.

[29] Kohda T, Asai A, Kuroiwa Y, Kobayashi S, Aisaka K, Nagashima G, Yoshida MC, Kondo Y, Kagiyama N, Kirino T, Kaneko-Ishino T, Ishino F (2001). Tumor suppressor activity of human imprinted gene PEG3 in a glioma cell line. Genes Cells.

[30] Feng W, Marquez RT, Lu Z, Liu J, Lu KH, Issa JP, Fishman DM, Yu Y, Bast RC. Jr. Imprinted tumor suppressor genes ARHI and PEG3 are the most frequently down-regulated in human ovarian cancers by loss of heterozygosity and promoter methylation. Cancer. 2008;112(7):1489–502. 10.1002/cncr.23323 18286529

[31] Ramsay RG, Gonda TJ (2008). MYB function in normal and cancer cells. Nat Rev Cancer.

[32] Introna M, Golay J (1999). How can oncogenic transcription factors cause cancer: a critical review of the myb story. Leukemia.

[33] Li L, Tan J, Zhang Y, Han N, Di X, Xiao T, Cheng S, Gao Y, Liu Y (2014). DLK1 promotes lung cancer cell invasion through upregulation of MMP9 expression depending on notch signaling. PLoS ONE.

[34] Bayramoglu A, Gunes HV, Metintas M, Değirmenci I, Mutlu F, Alatas F (2009). The association of MMP-9 enzyme activity, MMP-9 C1562T polymorphism, and MMP-2 and -9 and TIMP-1,-2,-3, and -4 gene expression in lung cancer. Genet Test Mol Biomarkers.

[35] Wang RJ, Wu P, Cai GX, Wang ZM, Xu Y, Peng JJ, Sheng WQ, Lu HF, Cai SJ (2014). Down-regulated MYH11 expression correlates with poor prognosis in stage II and III colorectal cancer. Asian Pac J Cancer Prev.

[36] Morrissey C, True LD, Roudier MP, Coleman IM, Hawley S, Nelson PS, Coleman R, Wang YC, Corey E, Langge PH, Higano CS, Vessella RL (2008). Differential expression of angiogenesis associated genes in prostate cancer bone, liver and lymph node metastases. Clin Exp Metastasis.

